# Ethical Orientation and Research Misconduct Among Business Researchers Under the Condition of Autonomy and Competition

**DOI:** 10.1007/s10551-022-05043-y

**Published:** 2022-01-29

**Authors:** Matthias Fink, Johannes Gartner, Rainer Harms, Isabella Hatak

**Affiliations:** 1 IFI Institute for Innovation Management, Johannes Kepler University Linz, Altenbergerstrasse 69, 4040 Linz, Austria; 2 Strategy, Collective Action and Technology Group, Grenoble Ecole de Management, 12, rue Pierre Sémard, 38000 Grenoble, France; 3 School of Economics and Management (SKJCE), Lund University, Box 117, 221 00 Lund, Sweden; 4 Faculty of Behavioural, Management and Social Sciences, University of Twente, Ravelijn 2109, P.O. Box 217, 7500 AE Enschede, The Netherlands; 5 Higher School of Economics, Laboratory for Economics of Innovation, Institute for Statistical Studies and Economics of Knowledge, HSE University, Myasnitskaya Ulitsa, 20, Moscow, Russian Federation 101000; 6 Swiss Institute of Small Business & Entrepreneurship (KMU-HSG), University of St. Gallen, Dufourstrasse 40a, 9000 St. Gallen, Switzerland

**Keywords:** Research misconduct, Ethical orientation, Deontological/consequentialist ethics, Autonomy, Competition, Survey, I23, K42, M14

## Abstract

The topics of ethical conduct and governance in academic research in the business field have attracted scientific and public attention. The concern is that research misconduct in organizations such as business schools and universities might result in practitioners, policymakers, and researchers grounding their decisions on biased research results. This study addresses ethical research misconduct by investigating whether the ethical orientation of business researchers is related to the likelihood of research misconduct, such as selective reporting of research findings. We distinguish between deontological and consequentialist ethical orientations and the competition between researchers and investigate the moderating role of their perceived autonomy. Based on global data collected from 1031 business scholars, we find that researchers with a strong deontological ethical orientation are less prone to misconduct. This effect is robust against different levels of perceived autonomy and competition. In contrast, researchers having a consequentialist ethical orientation is positively associated with misconduct in business research. High levels of competition in the research environment reinforce this effect. Our results reveal a potentially toxic combination comprising researchers with a strong consequentialist orientation who are embedded in highly competitive research environments. Our research calls for the development of ethical orientations grounded on maxims rather than anticipated consequences among researchers. We conclude that measures for ethical governance in business schools should consider the ethical orientation that underlies researchers’ decision-making and the organizational and institutional environment in which business researchers are embedded.

## Introduction

The threat of misconduct in the production of academic knowledge relevant to businesses (business research) has received increasing attention in recent years for reasons that include aggressive research funding and tenure situations, organizations striving to attain top academic standings, mounting public scorn with regard to impact, and researchers’ internal conflicts between personal and work values (Albrecht et al., 2011; Cabral-Cardoso, 2004; Dalen & Klamer, 2005; Fanelli et al., 2019; Hall & Martin, 2019; Martin, 2016; Moffatt, 2011; van Yperen et al., 2011). While blatant misconduct seems to be relatively rare, milder forms of misconduct in research—sometimes ascribed the euphemism *inappropriate conduct*—are relatively common. One study reports that every third junior researcher in engineering admits to selectively reporting results in research reports (Behrens & Gray, 2001). In this study, we focus on this milder form of research misconduct because, despite the relevance, ongoing concerns, and the publication of a few typologies categorizing business research misconduct and offering prescriptions for dealing with it, little empirical investigation has been undertaken. That lack of empirical research may result from the delicate nature of the topic, and respondents may be reluctant to have their ethics directly observed (Trevino, 1986). Experiments that manipulate research misconduct in real-life settings are difficult to justify.

Given data collection challenges in this delicate research area, empirical studies on research misconduct struggle to account for context fully. This is problematic as we need to understand how individuals interact with their organizational context to minimize misconduct in business research (Hall & Martin, 2019), which can be defined as the breach of maxims, standards, and rules of conduct (Taylor, 1975). Such understanding is central to the development of practical measures that ensure that research continues to contribute to the vital goal of science: Knowledge generation for the benefit of society (Mooken & Sugden, 2014; Stilgoe et al., 2013). Research misconduct, such as selective reporting, threatens the integrity of the scientific community (Cabral-Cardoso, 2004; Gilbert & Denison, 2003; Martinson et al., 2005) and can trap science in the *tragedy of the commons*, that is, that the pursuit of personal benefits that worsens the situation for all (Martin, 2012). In the worst case, practitioners, policymakers, and fellow researchers base their actions on research results biased as a result of misconduct (Boseley & Davey, 2020).

The research environment is characterized by rising levels of competition for publication opportunities, funds, and career opportunities (Fang & Casadevall, 2011; Hall & Martin, 2019). To redress “the dark side of the hypercompetitive environment of contemporary science” (Fang & Casadevall, 2011, p. 1012), the research community (Martin, 2013), the general public, and policymakers have called for research governance (Pandza & Ellwood, 2013). These calls led to the implementation of new external rules. Examples of external rules are codes of conduct such as the Code of Ethics of the Academy of Management (AoM) in 2006, ethical assessment procedures, and the formation of bodies that oversee adherence to those rules (Martin, 2013). However, as several recent misconduct cases uncovered only ex-post demonstrate, misconduct in business research cannot be eradicated even by applying the most rigorous rules and regulations (e.g., the Lancet’s retraction of a Covid study, Boseley & Davey, 2020).

Consequently, individual researchers’ ethical orientations, that is, the core logics that underpin their ethical reasoning (Reidenbach & Robin, 1990; Tanner et al., 2007), have become increasingly relevant in the discourse on how to promote ethical research. Consequently, universities have promoted ethical communities that inculcate moral socialization and mutual respect between faculty members by fostering an active discussion on ethical issues and acting on them (McCabe et al., 2001; Titus et al., 2008; Treviño & McCabe, 1994). While such initiatives can reinforce the ethical orientation among faculty, we still do not know whether ethical orientation can effectively reduce the threat of misconduct in research in general and particularly in business research, let alone which forms of orientation might do so. Following Eisenhardt et al. (2016) and Hall and Martin (2019), we focus on business research because it is a research area characterized by a pressure to secure publications in highly ranked journals and the significant rewards available to successful researchers (Hall & Martin, 2019). At the same time, published business research flows through to business practice. The last financial crisis made apparent the practical relevance of ensuring complete, unbiased, and independent business research.

Generally, ethical research is influenced by either a consequentialist ethical orientation that implies the evaluation of behavior in the face of its consequences or a deontological ethical orientation that implies the evaluation of behaviors drawing on the individual’s duties, rights, and obligations represented by maxims (Reidenbach & Robin, 1990; Tanner et al., 2007). At any point in the research process, researchers are, to some extent rooting their decisions in a consequentialist orientation and also in a deontological orientation, meaning every researcher is host to both ethical orientations. We argue that both types of ethical orientation, depending on their strength, restrain researcher misconduct in business research either through the threat of sanction or by potentially inculcating remorse, and that the effect is contingent upon the research context in terms of perceived autonomy and competition.

We apply ordinal logit regression to a global dataset collected from 1031 business scholars to empirically test two overarching research questions: Are business researchers with a particularly strong deontological or consequentialist ethical orientation less prone to research misconduct? Moreover, is the relationship between ethical orientation and research misconduct contingent on the competition and autonomy perceived by business researchers?

We find that researchers with a strong deontological ethical orientation are less prone to misconduct in business research. This effect is robust against different levels of perceived autonomy and competition. In contrast to our expectations, researchers’ consequentialist ethical orientation, in turn, is positively associated with misconduct in business research. Rising levels of competition in the researchers’ workplace reinforce this effect.

Our findings contribute to research and practice in several ways. First, we provide theoretically grounded arguments and empirical evidence on the relationship between the researchers’ ethical orientations and their research misconduct. By specifying the differences between deontological and consequentialist ethical orientations regarding research misconduct, we substantiate discussions about heterogeneity among researchers. Our results call for the development of ethical orientations founded on maxims rather than anticipated consequences among the research community. Second, by contextualizing our research by accounting for researchers’ perceptions of competition and autonomy—two core dimensions of the academic workplace—the findings signal the context-sensitivity of misconduct in business research. For those in practice, our findings highlight the potentially toxic combination of consequentialist researchers and the extent of their embeddedness in intensely competitive research environments. Knowing the empirical relevance of this potentially toxic combination raises questions about the personnel development policies frequently applied in business schools, such as having a group of junior researchers compete for one senior position. Our study highlights measures for ethical governance in business research that consider the ethical orientations that underlie researchers’ ethical decision-making and the organizational and institutional environment in which business researchers see themselves to be embedded.

## Conceptual Background and Hypotheses Development

### Deontological Versus Consequentialist Ethical Orientations and Research Misconduct

Given the significant potential impact, researchers must refrain from research misconduct (Kornfeld, 2012; Lund, 2000). Because rules and regulations have failed to curb research misconduct effectively, researchers’ ethical orientation has become important in the debate on ethical research (Boden et al., 2009). We differentiate between the deontological and consequentialist ethical orientations, which are rooted in two distinct foundations of ethical reasoning (Reidenbach & Robin, 1990; Tanner et al., 2007).

The deontological ethical orientation in the tradition of Kant (2003, p. 1788) builds on autonomous moral reasoning that is guided by the fit of a specific action with a person’s maxims (McNair, 2000). When a deontological ethical orientation is the foundation of moral reasoning, motivations for action are based on reason alone (O’Neill, 1989). Accordingly, a deontological ethical orientation is free from external constraints and is thus autonomous (Galvin, 1999). Accordingly, the categorical imperative (Kant, 2003, p. 1788) determines what a researcher ought to do. The categorical imperative uses a thought experiment to assess the moral quality of the principles (maxims) that individuals follow when determining their intentions: If those maxims are ones that the individual might want to become a generally valid law, the intention derived from the categorical imperative is, therefore, “good” (Schneewind, 1992). In the aforementioned thought experiment, the individual may consider the needs of the whole community by claiming a fictitious general validity and by including other people’s ends in his or her deliberations (Paton, 1962). It is not the apparent alignment between the motivation and the rules that is important, but the good intention (Korsgaard, 2002; Tyler & Blader, 2005). To clarify, most researchers commit themselves to the maxim of not reporting only selected results, even when to do so would be beneficial to them; they would refrain from suppressing results because they do not want such conduct to become generally accepted practice. Actions that diverge from maxims cause unpleasant remorse (Shalvi et al., 2011) and, therefore, a deontological ethical orientation reduces research misconduct.

#### H1

Researchers’ deontological ethical orientation is negatively related to research misconduct.

In practice, ethical considerations cannot be fully disentangled from the web of constraints (Schütz & Luckmann, 1973) in which researchers are embedded as members of the research community. Researchers with a consequentialist ethical orientation build their moral reasoning on the anticipated consequences of their actions, which are, in turn, determined by a web of constraints. Accordingly, their motivations are externally determined (see, the hypothetical imperative, Kant, 2003, p. 1788), for example, by legal regulations and codes of conduct that define what researchers ought to do (Luhmann, 1977) and which consequences they face if misconduct is proven. The defined consequences take effect within a community when the members accept the rule (Korsgaard, 1996) and control and sanction the misconduct of their peers (Eberl, 2004; Shalvi et al., 2011). For example, a generally accepted rule for empirical research within the research community would be that all study results should be reported, not a selection of them. Peers enforce this rule through control measures such as the peer-review process. Those researchers who break the rule face consequences, that can include the retraction of publications, the loss of their reputation, and their academic position. The anticipated negative consequences of breaches of the generally accepted rules of the game prevent researchers from acting in a manner that is contrary to the standards of the scientific community (Akaah, 1993). Accordingly, a consequentialist ethical orientation reduces research misconduct.

#### H2

Researchers’ consequentialist ethical orientation is negatively related to research misconduct.

### Research Misconduct Among Deontologists in the Context of Autonomy and Competition

We argue that the relationship between researchers’ deontological ethical orientation and research misconduct is contingent on perceived autonomy. Researchers have considerable autonomy when conducting their research. However, different researchers are likely to perceive the degree of autonomy in their environment differently, leading to a variation in their behavioral responses to it.

The level of autonomy researchers perceive determines how strongly their deontological ethical orientations affect the likelihood of research misconduct. High levels of perceived autonomy permit researchers to decide what, how, when, and with whom they want to work (Rose, 2001; Sax et al., 2002), thus providing them with academic freedom (Bland et al., 2005). Zhang et al., (2017, p. 236) state that autonomy makes researchers “feel self-determined and free from external controls or constraints” (on workplace autonomy, see also, Deci et al., 1989; Fricker & Schonlau, 2002; Spreitzer, 1995). Maxims induce researchers with strong deontological postures to justify their behavior to themselves rather than to others (Douglas, 2003). The more strongly researchers feel themselves to be autonomous, the more they can draw on their internal ethical standards as they make decisions (McNair, 2000; White, 2009). Accordingly, if researchers, who root their ethical research in a deontological ethical orientation, perceive themselves to be highly autonomous, maxims will limit their research misconduct even more than their peers with less perceived autonomy in the workplace. We, therefore, formulate the following hypotheses:

#### H3

The perceived level of autonomy moderates the relationship between researchers’ deontological ethical orientation and research misconduct, such that the negative relationship is stronger under high levels of perceived autonomy in the workplace.

While the perception of autonomy in the workplace reinforces the independence of researchers’ moral reasoning from influence by external forces (Debackere et al., 1995) and, thus, extends their freedom to follow their maxims, perceived competition affects the consequences of research misconduct. However, deontological moral reasoning builds on maxims rather than on the consequences of actions. The ethical value of the research maxims on which researchers with a deontological ethical orientation base their ethical reasoning is assessed independently from the causal constraints they face in the context they are embedded in (Kant, 2003). Accordingly, the impact of researchers’ deontological ethical orientation on research misconduct should not be contingent on the level of competition in the workplace. Therefore, we have not attempted to formulate a corresponding hypothesis.

### Research Misconduct of Consequentialists in the Context of Competition and Autonomy

The research environment is characterized by intense competition around publication opportunities, funding, and career opportunities (Fang & Casadevall, 2011; Hall & Martin, 2019). However, the perception of the degree of competition in the workplace is subjective, and different perceptions lead to variations in the behavioral response to the perceived competition. We argue that for researchers who base their moral reasoning on the anticipated consequences of their actions, perceived competition will influence how effectively their consequentialist ethical orientation deters research misconduct. This is because the behavior of researchers is influenced by their peers (Salancik & Pfeffer, 1978), who define expectations as the standard of peer-group comparisons (Brown et al., 1998) as well as sanctions and rewards for individual achievements (Williamson & Cable, 2003).

In intensely competitive work environments, researchers abstain from research misconduct because they anticipate that their peers are more likely to identify and report a cheating colleague, and, thus, cheats are less likely to prosper from cheating (Fink et al., 2012). While we see the merit in this whistle-blower argument, we argue that it downplays a more immediate effect of competition on the link between researchers’ consequentialist ethical orientation and research misconduct. The presence of intense competition in the first place implies that any gains obtained by cheating would be of greater relevance than in a less intense competitive environment (Shi et al., 2016). Such gains might include an extra publication, a publication in a more prestigious outlet, or extra funding from sponsors or funding agencies. Accordingly, high levels of competition reinforce the positive consequences of research misconduct anticipated by consequentialist researchers. Even if their peers monitor and report them more strictly in intensely competitive settings, the immediate competitive advantage of gains from research misconduct should outweigh the threat of negative consequences. We argue that a certain immediate gain from research misconduct is a stronger motivational factor than possible detriment owing to sanctions in the longer term (Åkerlund et al., 2016). Hence, intense competition should weaken the limiting effect of a consequentialist ethical orientation. These arguments lead us to propose:

#### H4

The perceived level of competition moderates the relationship between researchers’ consequentialist ethical orientation and research misconduct, such that the negative relationship is weaker under high levels of perceived competition.

Moreover, we assume that the extent to which anticipated consequences restrict research misconduct is not only contingent on the level of competition but also on the level of autonomy researchers perceive they have. This is because researchers who perceive they have a great deal of autonomy view their peers as having relatively little power to determine any negative consequences of their research misconduct; essentially, the chance of being sanctioned for ethical misconduct becomes less likely the greater autonomy a researcher accrues (Debackere et al., 1995). At the same time, high levels of autonomy do not reduce the positive reputational and career effects consequentialist researchers derive from the extra publications or extra funding resulting from their undetected and unsanctioned ethical misconduct. Hence, in researchers’ consequentialist ethical reasoning, ethical misconduct becomes less threatening with increasing autonomy, while the positive consequences remain intact. Because as part of their consequentialist orientation, researchers base their moral reasoning on the anticipated consequences of their action, increasing autonomy should, thus, weaken the limiting effect of their consequentialist ethical orientation on research misconduct. We therefore postulate:

#### H5

**Fig. 1:**
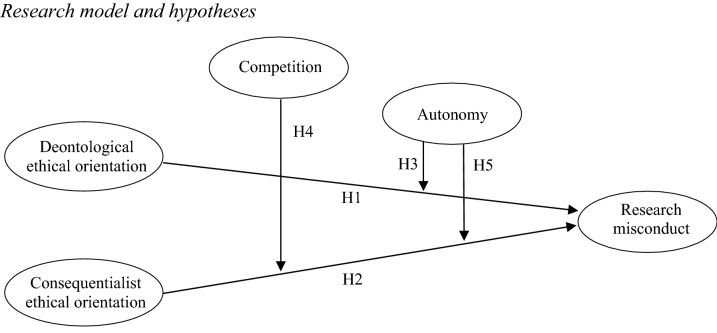
Research model and hypotheses

The perceived level of autonomy moderates the relationship between researchers’ consequentialist ethical orientation and research misconduct, such that the negative relationship is weaker under high levels of perceived autonomy. The five hypotheses are summarized in the theoretical model depicted in Fig. 1.

## Materials and Methods

### Data Collection

The sample informing this research is drawn from the worldwide population of management scholars, from doctoral students to full professors. We used contact details submitted to an e-mail contact list of active participants of the 2016 annual meeting of the AoM, the preeminent professional association for management scholars boasting a community of 20,000 scholars from 120 countries (AoM, 2021). Sampling among participants at an international science conference offers the opportunity to address a globally distributed population of a specific profession that has a specific research focus. That sampling approach has been successfully employed in empirical research, especially in the areas of health and medicine (Pezold et al., 2016; Zozus et al., 2017). Rigorous peer-review processes affecting all contributions to the AoM conference ensured that those involved were active research scholars who authored research presented at the conference. The individuals approached could thus be guaranteed to fall into our target group.

After a notification, we sent 10,716 e-mail invitations to all authors of papers presented at the 2016 AoM annual meeting, followed by two rounds of reminders seven and 14 days after the initial mailing. A web link pointed to a landing page where the participants could choose between a classic online survey or an interactive gamified survey. The gamified survey aimed to stir curiosity and thus enhance the response rate. The gamified survey was also offered an opportunity to check for social desirability bias (Georgiou et al., 2019; Stodel, 2015). Both survey versions contained the same formulations, sequence, and scales. Of the 2533 recipients who clicked the invitation link, 1684 completed the survey. We removed responses with missing values on the dependent variable and obtained a sample of 1031 individual responses to analyze. This response represents an effective response rate of 9.6%, which is slightly below the average of similar online survey studies (Pedersen & Nielsen, 2016; Petrovčič et al., 2016; Wouters et al., 2014). However, survey response rates have been decreasing significantly since 1986 (Sheehan, 2001) and are generally lower for web-based survey instruments (Blumenberg & Barros, 2018); Manfreda et al. (2008), for example, found an average response rate of 11% and a 6–15% confidence interval for online surveys. Lower response rates were recorded by studies that either randomly addressed members of professional associations (Poynton et al., 2019), used detailed questionnaires (Sauermann & Roach, 2013), or conducted studies on sensitive topics (Trautner et al., 2020).

We addressed potential nonresponse bias at the survey design stage by carefully designing the questionnaire to maintain the respondent’s interest, keeping it to a reasonable length, and establishing the importance of the study in the introductory e-mail (Yu & Cooper, 1983). We also assessed the analytic sample for potential nonresponse bias using two techniques to target specific types of nonresponse (Rogelberg & Stanton, 2007). First, we implemented the archival approach that compares the characteristics of the sample with those of the population. This approach is especially suited for identifying passive nonresponse, which results from external factors that keep recipients from returning the questionnaire on time. Passive nonresponse typically accounts for 85% of total nonresponse (Sosdian & Sharp, 1980). For our sample, we used the respondents’ age for archival analysis, as this variable has been shown to be relevant to research misconduct (Kelley et al., 1990) and was available for the members of the AoM. The comparison identified only a minor under-sampling of older researchers. Accordingly, passive nonresponse does not seem to be a significant concern. Second, we applied wave analysis, which involves comparing the results from early and late respondents, which is especially useful for controlling active nonresponse, which refers to conscious decisions not to participate in a study (Rogelberg et al., 2003). For our sample, the wave analysis did not identify any significant differences between early (first half) and late (second half) respondents, and hence we conclude that active nonresponse bias is also unlikely to be an issue in our sample.

Another potential threat to the validity of self-reported data in surveys on sensitive topics such as ethics is social desirability bias (Chung & Monroe, 2003; Cohen & Pant, 1998; Krumpal, 2013; Randall & Gibson, 1990). Social desirability bias emerges when respondents questioned about their compliance with widely accepted behavioral norms over-report behavior that is in line with those norms and under-report deviations from them (Podsakoff et al., 2013). Because the effectiveness of social desirability scales or single items in directly measuring and correcting such bias is heavily debated, and such strategies may even introduce a systematic error (Fisher, 1993; Kam, 2013; Larson, 2019; Paulhus, 2002), we decided to implement a mix of measures in the design of the data collection that has been found to mitigate the threat of social desirability bias. Notifying all meeting participants that they would be invited to participate in a survey (DeLeeuw, 2018), the anonymity guaranteed to all respondents, and the avoidance of personal contact by opting for a self-administered web-based survey (Hunter, 2012; Moy & Murphy, 2016; Richman et al., 1999; Tourangeau & Yan, 2007) should have mitigated social desirability bias (Couper, 2017). Additionally, for those respondents who opted for the gamified version of the survey, the perception of the power relation between researcher and respondent (Denzin, 1989) and the feeling of being evaluated (Armstrong et al., 2016) should have been further ameliorated because playing a game creates a cognitive load that reduces respondents’ concern over social desirability. Accordingly, gamified surveys are less prone to faking and distortion (Georgiou et al., 2019; Stodel, 2015). Comparing the values of the dependent and independent variables collected via the traditional survey to those collected via the gamified survey revealed no significant differences. Because any social desirability bias would be stronger in the subsample collected via the traditional web survey than in the subsample collected via gamified survey, the lack of significant differences is an indication that social desirability bias is not an issue in our sample.

### Variables

#### Dependent and Independent Variables

To capture the dependent and independent variables, we used the vignette technique (Finch, 1987; Hyman & Steiner, 1996) that is well-established in business ethics research (Hox et al., 1991). For research on ethical orientations and ethical behavior, the vignette technique offers several advantages over direct-question-based measures, including greater realism better approximates real-life decision-making (Barnett et al., 1994; Cavanagh & Fritzsche, 1985; Robertson, 1993), enhanced internal validity, measurement reliability, and ease of replication through standardized stimuli provided to all respondents (Lysonski & Gaidis, 1991; Weber, 1992), enhanced construct validity through a more rigid focus on specific aspects of the phenomenon under research (Cavanagh & Fritzsche, 1985; Weber, 1992) and the possibility to differentiate between ethical principles and behavior (Cavanagh & Fritzsche, 1985).

Following the constant-variable-value vignette method (Cavanagh & Fritzsche, 1985), all respondents received the same description (vignette) of an ethical dilemma in a research situation (Table 1): A client asks the researcher to suppress undesirable results in a study conducted for the client (Loo, 2002). The practical relevance of this setting is highlighted by Behrens and Gray (2001), who reports that 35% of researchers allow their industry partners to delete content in research reports, a practice that can be deemed unethical. The respondents had to decide whether to suppress results. The scale was anchored with “not likely at all [suppress]” (1) and “very likely [suppress]” (6). The frequencies per category were: 1 (not likely at all): 357 (34.6%); 2: 337 (32.7%); 3: 162 (15.7%); 4: 101 (9.8%); 5: 56 (5.4%), 6 (very likely): 18 (1.8%).

**Table 1:** Descriptive statistics and operationalization

	Mean	SD	Min	Max
Research misconduct: suppression of research results upon client’s request *Vignette: Please imagine the following situation and answer the questions below honestly. You finalized a research project for an external client. However, your client is not satisfied with some of the results. The satisfaction of your client is very important to you because you need the client’s third-party funding for your next research project. He asks you to visit him in his office to talk about the next steps. In this meeting, your client asks you to exclude some of your results from the final report* *We then asked the responded to indicate how likely they follow their clients request to report only selected results that are favorable for their clients. Verbal anchors: 1* = *not likely at all, 6* = *very likely. Note: Due to low frequencies, the categories 4, 5, and 6 were combined into one that denotes a high likelihood of agreeing to suppress the unfavorable results. Accordingly, ‘research misconduct’ consists of four ordered categories*	1.85	1.08	1	4
Consequentialist ethical orientation *Item 1: I chose this option because this option can be justified by the consequences. Item 2: I chose this option because the outcomes of the chosen option produce the best net value. Item 3: I chose this option because the positive outcomes outweigh the negative consequences. Item 4: I chose this option because cost–benefit analysis makes sense with this topic*	3.67	1.30	1	6
Deontological ethical orientation *Item 1: I chose this option because this option can be justified by the consequences. Item 2: I chose this option because the outcomes of the chosen option produce the best net value. Item 3: I chose this option because the positive outcomes outweigh the negative consequences. Item 4: I chose this option because cost–benefit analysis makes sense with this topic*	4.46	1.19	1	6
Autonomy at work *Item 1: It is my decision which topic I research. Item 2: It is my decision how I organize my work. Item 3: In my research, I can launch my own initiatives. Item 4: I can make decisions independently regarding my research. Item 5: In my research, I can make decisions without obtaining the consent of others. Item 6: Which methods I apply in my research is my decision. Item 7: I am free to choose how I meet my targets in my research*	5.03	0.83	1.29	6
Competition at work *Item 1: Generally, the competition between me and other researchers is fierce. Item 2: Within my organization, the competition between me and other researchers is fierce. Item 3: There is fierce competition between me and other researchers for research positions. Item 4: There is fierce competition between me and other researchers for research funding. Item 5: I am under huge pressure to perform better than others at my career level*	3.82	1.16	1	6
Woman (base: man)	0.48		0	1
Number of papers published in refereed journals in the last 3 years	4.40	4.80	0	45
Amount of funding obtained for research in the last 3 years				
10,000 USD or less (base)	0.56		0	1
10,001–100,000 USD	0.21		0	1
100,001–300,000 USD	0.11		0	1
> 300,000 USD	0.12		0	1
Current position				
Ph.D. student	0.25		0	1
Postdoc	0.08		0	1
Assistant professor	0.25		0	1
Associate professor	0.19		0	1
Full professor (base)	0.22		0	1
Private university (base: public university or research institute)	0.21		0	1
Promoted in last 3 years (base: not promoted)	0.31		0	1
Gamified survey (base: conventional survey)	0.73		0	1

1031 observations

*SD*  standard deviation

Due to low frequencies in the top three categories, we combined those categories into one that denotes a high likelihood of agreeing to suppress the unfavorable results. Accordingly, the *dependent variable* that we call *research misconduct* consists of four ordered categories.

Following Tanner et al. (2007), we asked the respondents to evaluate the relevance of *consequentialist* and *deontological ethical orientations* for the decision they just made, which form our *independent variables*. Ethical orientations are rather stable and guide decisions across different life spheres (Reidenbach & Robin, 1990). Using the vignette technique, we assume that the moral foundations that the respondents report in the hypothetical decision-making scenario are ones that also guide them in other similar research-based decisions (Barnett et al., 1994; Cavanagh & Fritzsche, 1985). We used three items each to capture the reasons reflecting consequentialist and deontological ethical orientations (Table 1) adapted from Reidenbach and Robin (1990) and Loo (2002. An example of an item measuring a consequentialist ethical orientation is “I chose this option because the outcomes produce the best net value.” In contrast, an example of a deontological ethical orientation is “I chose this option because some behaviors are definitely right or wrong, irrespective of the consequences.” All items were coded so that higher numbers indicate a higher score on the construct.

#### Moderating Variables

The first moderator, *autonomy*, describes the degree to which researchers feel that they are free to make their own decisions regarding their research. The second moderator, *competition*, describes the degree to which researchers feel exposed to fighting with their peers over career opportunities and funds. We measured autonomy with a seven-item scale adapted from Morgeson and Humphrey’s (2006) work design questionnaire and competition with a five-item scale (Table 1) adapted from Fink et al. (2012). For each item, respondents indicated their degree of perceived autonomy/competition on a 6-point rating scale where high values denote high levels of autonomy/competition.

#### Control Variables

First, we controlled for the respondents’ *research productivity* using two variables: the number of publications and the amount of research funding obtained in the past three years. Although measuring researchers’ productivity remains a thorny issue (van Noorden, 2010; Wootton, 2013), there is consensus that publications and the amount of research funding are key output measures (Garcia & Sanz-Menéndez, 2005; Gulbrandsen & Smeby, 2005; Linton et al., 2012). We measured the number of publications with an open question and the funding obtained on an ordinal scale (Table 1). Given the skewness of the publication measure, we used its natural logarithm in the regression analysis. Next, we controlled for the respondent’s *biological sex* because women have been reported to be more sensitive to ethical issues than men (Glover et al., 2002; Kelley et al., 1990; O’Fallon & Butterfield, 2005).

Moreover, we included the respondent’s *academic career stage* as a further control because the career stage may influence perceived competitive pressure (see, e.g., Hatak et al., 2015 on socio-emotional selectivity theory) and perceived autonomy (Dowd & Kaplan, 2005). The academic career stage can also influence a researcher’s inclination to ethical misconduct (Fanelli et al., 2015). Because the individual’s age correlates strongly with their position on the academic career ladder, we omitted age from the control variables. Additionally, we controlled for whether the respondent works in a *private* university or research institute (versus a public one) and whether they have been *promoted* in the last three years. Private and public universities differ in the level of competition and autonomy (Estermann et al., 2011; Ivory & Shipton, 2020). A recent promotion reduces the competitive pressure and limits the immediate positive consequences of ethical misconduct for the career. Finally, we controlled for the *type of survey* the respondent opted for (conventional or gamified) because different online data collection methods may affect the responses (Keusch & Zhang, 2017).

### Discriminant Validity

Before computing index scores of the multi-item scales for the subsequent regression analysis, we examined their discriminant validity using confirmatory factor analysis. Specifically, we compared a specification where all items load on their theoretically intended factors to ones where two sets of items load on a single factor, while the other two load on their own factors, and a specification where all items load on a single factor. In each case, the theoretically intended model resulted in a superior fit with the data. Indeed, the model where all items load on their intended factors shows a good fit with the data (Hu & Bentler, 1999): the comparative fit index (CFI) score is 0.976 (recommended minimum 0.95), the standardized root mean squared residual (SRMR) index value is 0.035 (recommended maximum 0.08). The root mean squared error of approximation (RMSEA) is 0.040 (recommended maximum 0.06). Furthermore, the model’s average variance extracted (AVE) scores exceed the squared correlations among the latent variables, which provides further evidence for satisfactory discriminant validity (Fornell & Larcker, 1981).

### Common Method Bias

Scholars have highlighted the threat of *common method bias* (CMB) to empirical research relying on cross-sectional data (Lindell & Whitney, 2001), and especially to the most common self-report surveys with cognitions as dependent and independent variables (Harrison et al., 1996). Several ex-ante and ex-post measures can be applied to address the risk of CMB (Podsakoff et al., 2003). However, Spector (2006) and Richardson et al. (2009) have provided substantial evidence showing that ex-post statistical measures to adjust analyses for CMB are unreliable and often misleading. Accordingly, we employed the recommended strategies to avoid CMB ex-ante. First, we protected the respondents’ anonymity to mitigate evaluation apprehension (Podsakoff et al., 2003). Second, we used different question formats and randomized the order of scales in the questionnaire.

Finally, we checked the data for potential bias due to this study's survey strategy. A comparison on the descriptive level of the data collected with the gamified and traditional surveys shows that while the gamified survey attracted a slightly younger set of respondents and one with slightly more women, only the level of autonomy is significantly higher in the sample drawn from the traditional survey. The response rate did not differ between the two types of the survey instrument. Accordingly, bias stemming from the survey strategy does not appear to affect our study.

### Analysis Strategy and Diagnostics

The ordinal nature of the dependent variable prompted us to choose ordinal logit regression as the most appropriate statistical modeling technique. We compared the Akaike and Bayesian information criteria between an ordinal and a linear regression model. The criteria favored the ordinal specification. We examined the models for multicollinearity, influential observations, and the parallel regression assumption that underlies ordinal regression models before performing the final estimations.

The mean variance inflation factor (VIF) score was 1.40, and the highest VIF, which pertained to the category Ph.D. student in the academic career stage variable, was 3.01. These are below the conventional threshold of 10 for multicollinearity.

We analyzed potentially influential observations by computing residuals, leverages, and Cook’s distance statistics, as well as examining plots of squared residuals and leverages. We identified 14 observations that had potentially influenced the model estimates and excluded them from the final analysis sample, which thus became 1031 observations.

The parallel regression assumption that underlies ordinal regression models maintains that the relationship between each pair of outcome groups is the same; that is, a single set of coefficients applies to all outcome groups. If the model violates this assumption, a generalized model where one or more coefficients vary between different outcome groups must be estimated. To test this assumption, we first compared a model specification where the parallel-lines constraint is imposed on all variables with a model where the constraint is relaxed for all variables. We found that the parallel regression assumption did not hold for our model. We then followed an iterative process of relaxing the parallel-lines constraint for one variable at a time (Williams, 2006) to determine which variables violate the assumption. These tests revealed that the coefficients of both independent variables and two control variables (promoted in the last three years and the assistant professor category in the academic career stage) need to be estimated separately for each pair of outcomes. Therefore, the final regression models use a generalized ordinal logit specification where we apply the parallel regression constraint to all variables except for the four mentioned above.

### Descriptive Statistics

Table 1 shows the means, standard deviations, minima, and maxima for all variables used in the analysis. Table 2 displays the correlation matrix. The dependent variable and two control variables (research funding and current position) are ordinal rather than continuous, so we report Spearman’s rank-order correlations. We do not detect any unexpected correlations (Fig. 1).

**Table 2:** Correlations

	1	2	3	4	5	6	7	8	9	10	11
1. Research misconduct	1										
2. Consequentialist	0.19	1									
3. Deontological	− 0.61	− 0.00	1								
4. Autonomy	− 0.24	− 0.04	0.21	1							
5. Competition	0.14	0.07	− 0.10	− 0.25	1						
6. Woman	0.07	0.03	− 0.03	− 0.15	0.09	1					
7. Number of papers (log)	− 0.11	0.00	0.07	0.32	0.02	− 0.18	1				
8. Research funding	− 0.06	− 0.03	0.01	0.09	0.03	− 0.07	0.35	1			
9. Current position	− 0.22	− 0.04	0.13	0.44	− 0.17	− 0.20	0.64	0.27	1		
10. Private university	− 0.08	− 0.03	0.06	0.08	− 0.07	0.01	0.03	− 0.07	0.09	1	
11. Promoted in last 3 years	0.05	0.02	− 0.01	0.05	0.05	− 0.02	0.20	0.16	0.18	0.01	1
12. Gamified survey	0.04	0.04	− 0.05	− 0.12	0.01	0.12	− 0.14	0.00	− 0.15	0.00	0.06

1031 observations. Spearman rank-order correlation coefficients

## Results

Table 3 presents the results of the generalized ordinal logit regression models. Model 1 presents the unconditional effects of all variables (Hypotheses 1 and 2), whereas Model 2 adds the interaction terms (Hypotheses 3–5). Note that Table 3 displays a single coefficient across all three thresholds for variables for which the parallel regression assumption holds, and the corresponding restriction is applied. The thresholds are dichotomizations of the ordinal variable into binary outcomes, such as those analyzed in logistic regressions. For example, at the third threshold, we compare the first three categories of the dependent variable categories against category 4. A different coefficient is displayed for each threshold for the four variables (and the interaction terms they are included in) that violate the parallel regression assumption. In addition to the logic coefficient, Table 3 displays the standard error, the *p* value, and the odds ratio as an effect size estimate.

**Table 3:** Generalized ordinal logit regression results pertaining to research misconduct

	*Model 1* Logit coefficient (SE) *p* [OR]			*Model 2* Logit coefficient (SE) *p* [OR]		
	Threshold 1	Threshold 2	Threshold 3	Threshold 1	Threshold 2	Threshold 3
Consequentialist	0.24 (0.06) *0.001* [1.27]	0.41 (0.07) *0.001* [1.51]	0.86 (0.11) *0.001* [2.33]	0.24 (0.06) *0.001* [1.27]	0.40 (0.07) *0.001* [1.50]	0.89 (0.11) *0.001* [2.43]
Deontological	− 1.12 (0.09) *0.001* [0.32]	− 1.28 (0.09) *0.001* [0.28]	− 1.47 (0.11) *0.001* [0.23]	− 1.10 (0.09) *0.001* [0.33]	− 1.28 (0.09) *0.001* [0.28]	− 1.50 (0.11) *0.001* [0.22]
Autonomy	− 0.24 (0.09) *0.006* [0.79]			− 0.23 (0.10) *0.019* [0.79]		
Competition	0.08 (0.06) *0.154* [1.08]			0.02 (0.07) *0.819* [1.02]		
Woman (base: man)	− 0.01 (0.13) *0.931* [0.99]			0.00 (0.13) *0.984* [1.01]		
Number of papers (log)	0.14 (0.10) *0.195* [1.15]			0.15 (0.11) *0.151* [1.16]		
Research funding (base: 10,000 USD or less)						
10,001–100,000 USD	− 0.23 (0.16) *0.154* [0.79]			− 0.24 (0.16) *0.143* [0.79]		
100,001–300,000 USD	− 0.20 (0.21) *0.344* [0.82]			− 0.20 (0.21) *0.337* [0.82]		
> 300,000 USD	− 0.42 (0.21) *0.050* [0.66]			− 0.44 (0.21) *0.040* [0.65]		
Current position (base: full professor)						
PhD student	0.85 (0.25) *0.001* [2.35]			0.86 (0.25) *0.001* [2.37]		
Postdoc	0.74 (0.27) *0.013* [2.10]			0.75 (0.27) *0.006* [2.13]		
Assistant professor	0.30 (0.22) *0.170* [1.34]	0.85 (0.25) *0.001* [2.35]	0.60 (0.28) *0.034* [1.82]	0.32 (0.22) *0.141* [1.38]	0.82 (0.23) *0.001* [2.27]	0.50 (0.29) *0.083* [1.65]
Associate professor	0.04 (0.20) *0.839* [1.04]			0.04 (0.21) *0.835* [1.04]		
Private university	1.210.22 (0.15) *0.145* [0.80]			− 0.24 (0.15) *0.122* [0.79]		
Promoted in last 3 years	0.30 (0.17) *0.078* [1.35]	0.58 (0.18) *0.001* [1.78]	0.09 (0.23) *0.702* [1.09]	0.31 (0.17) *0.068* [1.37]	0.59 (0.18) *0.001* [1.81]	0.06 (0.23) *0.807* [1.06]
Gamified survey	− 0.06 (0.14) *0.696* [0.94]			− 0.06 (0.15) *0.677* [0.94]		
Interactions						
Consequentialist × autonomy				0.01 (0.08) *0.869* [1.01]	0.15 (0.09) *0.106* [1.16]	0.17 (0.10) *0.103* [1.19]
Consequentialist × competition				0.02 (0.05) *0.722* [1.02]	0.04 (0.06) *0.564* [1.04]	0.19 (0.09) *0.030* [1.21]
Deontological × autonomy				− 0.14 (0.12) *0.255* [0.87]	− 0.07 (0.11) *0.496* [0.93]	0.01 (0.11) *0.962* [1.01]
Deontological × competition				0.03 (0.07) *0.641* [1.03]	− 0.10 (0.07) *0.126* [0.90]	− 0.09 (0.07) *0.211* [0.91]
Constant	0.57 (0.28) *0.045* [1.76]	− 1.78 (0.29) *0.001* [0.17]	− 3.23 (0.33) *0.001* [0.04]	0.54 (0.29) *0.060* [1.71]	− 1.79 (0.29) *0.001* [0.17]	− 3.26 (0.34) *0.001* [0.04]
McFadden pseudo *R*^2^	0.24			0.24		
Nagelkerke pseudo *R*^2^	0.50			0.51		
Log likelihood	− 1035.99			− 1030.92		

1031 observations

*SE* standard error, *OR* odds ratio

Threshold-specific estimates are reported only for those variables that violate the proportional odds assumption. Threshold 1 contrasts category 1 of the dependent variable (absolutely would not suppress unwelcome research results) with the higher categories 2 (most likely would not suppress), 3 (perhaps would suppress), and 4 (most likely would suppress); Threshold 2 contrasts categories 1 and 2 with categories 3 and 4; and Threshold 3 contrasts categories 1, 2, and 3 with category 4

Model 1 shows that both types of ethical orientation are significantly related to the dependent variable. A deontological ethical orientation is negatively and significantly associated with research misconduct. This finding supports Hypothesis 1. In contrast, a consequentialist ethical orientation has a positive and significant association with research misconduct, that is, the likelihood of agreeing to suppress unwelcome research results. This finding does not support Hypothesis 2 suggesting a negative effect.

Interestingly, the association between consequentialist ethical orientation and research misconduct is particularly strong. The respondents show the highest likelihood of agreeing to suppress unfavorable results. The odds ratios for the highest threshold in the outcome variable are 2.33 for consequentialist and 0.23 for deontological ethical orientation. The odds ratios of positive and negative odds are difficult to compare because negative coefficients result in odds ratios of less than one. To make them comparable with positive odds ratios, they can be reversed (1/odds ratio). The reverse odds ratio for deontological ethical orientation is 4.35, which is greater than the odds ratio of consequentialist ethical orientation.

Model 2 adds the interaction terms to the equation. For reasons of parsimony, we include all interaction terms in a single model. We also tested the interactions separately and found the coefficients to be robust. Therefore, the only significant interaction in the model is the one between consequentialist ethical orientation and competition. The interaction term has a positive sign, which suggests that the positive effect of a consequentialist ethical orientation on high levels of research misconduct becomes stronger when the level of competition increases. This result is significant at a level of 0.05 for the third threshold, which is when we compare the first three categories of the dependent variable against the fourth category.

**Fig. 2:**
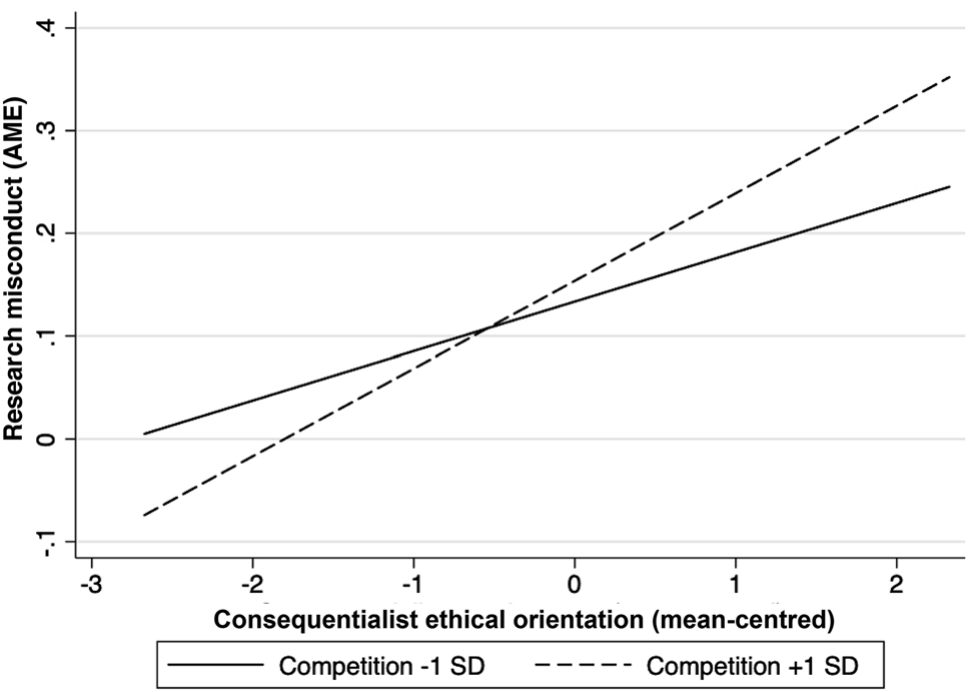
Average marginal effects of consequentialist ethical orientation on research misconduct at different levels of competition. *Notes:* The average marginal effect of consequentialist ethical orientation on fraudulent behavior is 0.05 (*p* < 0.001) when the competition level is 1 SD below its mean, whereas it is 0.09 (*p* < .001) when the competition is 1 SD above its mean

To illuminate the interaction effect, we computed the average marginal efffect (AME) of a consequentialist ethical orientation on the highest level of research misconduct with the moderator set one standard deviation unit below and above its mean (Ai & Norton, 2003; Aiken & West, 1991). We computed the AME on all levels, but—for simplicity’s sake—we display only the highest level on the ordinal scale of the dependent variable in this illustrate on. The y-axis depicts the average marginal effect (AME). Figure 2 shows that the effect of a consequentialist ethical orientation on the highest level of research misconduct is stronger (AME = 0.09, *p* = 0.000) when the competition is one standard deviation unit above its mean than when the level of competition is one standard deviation unit below its mean (AME = 0.05, *p* = 0.000). These findings support Hypothesis 4 but not Hypotheses 3 and 5.

## Discussion

This study investigated whether business researchers who base their ethical reasoning on a consequentialist and deontological ethical orientation are effectively protected from the temptation of research misconduct and what role context plays. We expected both types of researchers’ ethical orientations to be associated with less research misconduct. We also expected the relationship between researchers’ ethical orientation and research misconduct to be contingent on perceived competition and autonomy. Our results indicate that a strong deontological ethical orientation restricts research misconduct, while a strong consequentialist ethical orientation fosters misconduct in the business research context. This finding may be explained by the logic researchers follow in their consequentialist reasoning: They balance the potential costs and benefits of their behavior. If the perceived benefits are high and the expected costs of detection are low, research misconduct is attractive for those who act upon the expected consequences of their behavior. This logic is in line with cost-benefits arguments developed in legal theories of misconduct, which argue that the propensity of individuals to engage in misconduct is a function of the likelihood of detection and punishment compared with the utility gained from the misconduct (Becker, 1968; Hornuf & Haas, 2014).

Our findings show that the level of competition in the workplace is a factor in research misconduct among consequentialist researchers. Note that this effect may be small but is significant, supporting the notion of time-discounting related to the benefits and costs of misconduct (Åkerlund et al., 2016). Time-discounting means that immediate benefits are more relevant in moral reasoning than subsequent sanctions. Moreover, these punishments might not ultimately be so severe, as empirical evidence on the subsequent careers of researchers found guilty of misconduct shows (Galbraith, 2017). In summary, researchers with a strong consequentialist ethical orientation working in highly competitive settings may constitute a potentially toxic configuration.

We contribute to theory as follows: A cost–benefit perspective in research on misconduct (Greve et al., 2010; Hall & Martin, 2019) can fall short in considering individual attributes that go beyond rational choice arguments. We do not dispute the existence of a rational posture toward ethical research, nor do we dispute that a consequentialist ethical orientation has a strong effect on negative outcomes. Instead, this research highlights the importance of considering heterogeneity in ethical orientations and researchers’ individual attributes. We build on and integrate research from philosophy, psychology, and sociology that has focused on unfolding individual attributes while accounting for social contexts to explain differences in behavior. Our research clearly illustrates the importance of explicitly including ethical orientation in theory in that it offers evidence of the effects deontological and consequentialist ethical orientations exert on research misconduct.

Interestingly, deontological orientations seem to reduce research misconduct far more than consequentialist orientations increase it. In line with self-regulation theory (Bandura, 1991), this suggests that self-regulation is more effective than sanctions in reducing misconduct in business research. For researchers to refrain from misconduct, self-inflicted remorse seems to be more relevant than the punishment imposed by others. A possible explanation might be that in deontological reasoning, remorse is inevitable, while consequentialist reasoning encourages the hope of escaping punishment because the research community might fail to detect and punish the misconduct. Researchers have adopted various perspectives on rules and regulations when investigating the forms of ethical (mis)conduct (Hall & Martin, 2019). Our study extends these efforts by suggesting that focusing on the variations in ethical orientations offers attractive avenues for explaining research misconduct.

Second, we contribute a contingency perspective on research misconduct. The episodic nature of research misconduct makes it likely that individual attributes are not its only stable antecedents. We find that perceived competition matters in the positive relationship between a consequentialist ethical orientation and research misconduct. Intense competition not only erodes the limiting effect of a consequentialist ethical orientation on researchers’ ethical misconduct but even turns it negative. Accordingly, under the condition of low to moderate levels of competition, stronger consequentialist ethical orientations still restrict researchers’ ethical misconduct. However, when competition in the workplace is fierce, that misconduct is more likely if the researchers’ moral reasoning is built on the consequences of their actions. This finding aligns with the general strain perspective (Agnew, 1992) that postulates that when individuals face stressful circumstances concerning the present or the future, they experience negative emotions (Rauch et al., 2018). To alleviate them, individuals engage in adaptive behaviors, including questionable behavior (Agnew, 1992). Not publishing a much-needed paper or attracting research funding because of excessive competition may constitute such a source of strain for consequentialists because they will be deprived of essential resources. General strain theory postulates that external conditions influence whether individuals respond to the strain with acceptable or questionable behavior, for example, through research misconduct (Lewellyn et al., 2017). We extend this stream of research (O’Boyle et al., 2017) by showing that for research misconduct, specific aspects of the work context (i.e., competition) are relevant for consequentialists rather than for deontological reasoning among researchers. This insight adds nuance to the general strain perspective.

The current research has implications for practical research governance. First, ethics training programs for researchers are widespread and are becoming more effective (Watts et al., 2017). While Watts et al. (2017) suggest that participant demographics influence the effectiveness of ethics training for the sciences (see Medeiros et al., 2017 for business ethics), we add that differences in ethical orientation may influence research misconduct too; for example, documentation of negative consequences of research misconduct may tip the consequentialists’ scale in favor of ethical research. In light of our findings, the display of ethical role models in research might become an essential aspect of ethics training programs in business schools and universities to reinforce deontological researchers’ maxims. However, consensus on the most effective forms of ethics education is just beginning to emerge (Medeiros et al., 2017), and research has not yet addressed the difference in ethical orientations among scholars.

Second, because perceived competition increases the adverse effects of a consequentialist ethical orientation on ethical research, leaders of academic units concerned with business research may want to reduce the intensity of competition. First, management could include teaching and service for the faculty and the scientific community as performance criteria to reduce the importance of research publications and the competitive pressure related to them. Second, management could de-emphasize journal-based output metrics and “assess research on its own merits rather than on the basis of the journal in which the research is published” (DORA, 2020). That action could reduce the competition for artificially scarce spots in top journals. Third, management could reward transparent (Whetstone & Moulaison-Sandy, 2020) collaborative research to counter the winner-takes-all mentality that is implicit in the (over-) emphasis on first authorship (Floyd et al., 1994; Krasnova et al., 2012). Finally, management could reduce overall work-related stress in academia (Urbina-Garcia, 2020) because those who cope better with competition-induced stressors may be less likely to resort to research misconduct.

Our study has limitations. First, the sample is limited to business research scholars. Researchers from China could not be reached because the Chinese authorities’ firewall blocked our invitation e-mail. Accordingly, we refer to a specific discipline and its research culture alone. To address this limitation, we suggest extending this research to other research fields and harvesting the Chinese perspective by sending invitations to academics to complete the survey from within the country. Because we guaranteed the respondents anonymity, we could not control for differences between the geographical locations of the researchers. However, the relationship between researchers’ ethical orientation and research misconduct, as well as whether it is contingent on autonomy and competition, may differ depending on the country context or the discipline. Neither did we account for different types of research approaches and research institutions. It could be argued that business researchers working with secondary data would consider research misconduct differently than those who engage in field studies in partnership with companies and managers. Also, ethical research may be easier to safeguard in natural sciences than in social sciences because empirical research investigating natural laws can be challenged by reproducibility (Fink et al., 2012). It is more difficult to reproduce findings in social sciences, such as business research, as the exact social situation is difficult to replicate.

Second, this study does not investigate the antecedents of researchers’ ethical orientation. Extending the model with those antecedents could enable the authors of future studies to formulate even more specific recommendations for reducing the threat of ethical misconduct in business research.

Third, while the relationship between deontology and misconduct is strong, the effect of the interaction between consequentialism and perceived competition is, although significant, less strong. Future research is needed to explore this interaction in more detail. For example, in addition to the perceived competition in terms of degree, the way in which researchers frame the competition they face in the workplace (Ryckman et al., 1996) may influence the effect of the interaction between consequentialism and perceived competition.

Fourth, our study is limited to one type of research misconduct: the falsification of results through the suppression of undesired responses. While this is a common (Behrens & Gray, 2001) and harmful practice (Chalmers, 1990), there are other types of research misconduct, such as those from the widely used FFP (fabrication, falsification, plagiarism) typology (Resnik et al., 2015). To address this limitation, we suggest extending research to these other types of misconduct (Hall & Martin, 2019). Doing so would, however, be challenging for those types of misconduct based on active deeds such as the fabrication of research data and results (the sin of commission) rather than suppressing undesired responses (the sin of omission), for example. This is because people judge acts of omission as less immoral than acts of commission (Spranca et al., 1991), which might bias the self-reporting of such behavior. Moreover, the purview of this study is limited to contract research for external clients. We call for replication of our study with vignettes covering fundamental research. Such vignettes could, for example, confront respondents with the need to decide whether or not to report rejected hypotheses in their submissions to scientific journals, knowing that papers including rejected hypotheses are less likely to be accepted for publication.

Future research could also investigate other moderators of the relationship between ethical orientation and research misconduct. For example, the academic workload is high worldwide (Shin & Jung, 2014), which can encourage unethical research (Schwepker & Good, 2017; Yam et al., 2014). The effect of an extensive workload and work pressure on researchers’ ethical conduct remains an attractive area of future inquiry. In addition, Covid-19 and its implications for the future of work affect academia in many ways. A salient point for the topic of unethical behavior may be the increased time spent working from home. Researchers working primarily in that manner may feel their work is less supervised and hence feel greater autonomy in their research. To the extent that this situation reduces the consequentialists’ expected likelihood of detection, it could increase research misconduct. Again, future research might test that possibility and which interventions could help researchers cope with stress in the new teleworking environment.

We conclude that researchers with a strong deontologist ethical orientation are less prone to engage in research misconduct, and they are also likely to have a more robust attitude in terms of autonomy and in the face of competition. Researchers with a strong consequentialist ethical orientation are more inclined toward research misconduct. However, the finding that competition influences the link between a consequentialist ethical orientation and research misconduct in business research can also be a stimulus to design academic workspaces that mitigate competitive pressures for those researchers.
